# Homogenization of bacterial plastisphere community in soil: a continental-scale microcosm study

**DOI:** 10.1093/ismeco/ycad012

**Published:** 2024-01-10

**Authors:** Yuanze Sun, Mochen Wu, Siyuan Xie, Jingxi Zang, Xiang Wang, Yuyi Yang, Changchao Li, Jie Wang

**Affiliations:** Beijing Key Laboratory of Farmland Soil Pollution Prevention and Remediation, Department of Environmental Science and Engineering, College of Resources and Environmental Sciences, China Agricultural University, Beijing 100193, China; Beijing Key Laboratory of Farmland Soil Pollution Prevention and Remediation, Department of Environmental Science and Engineering, College of Resources and Environmental Sciences, China Agricultural University, Beijing 100193, China; Beijing Key Laboratory of Farmland Soil Pollution Prevention and Remediation, Department of Environmental Science and Engineering, College of Resources and Environmental Sciences, China Agricultural University, Beijing 100193, China; Beijing Key Laboratory of Farmland Soil Pollution Prevention and Remediation, Department of Environmental Science and Engineering, College of Resources and Environmental Sciences, China Agricultural University, Beijing 100193, China; Key Laboratory of Arable Land Conservation (North China), Department of Soil and Water Science, College of Land Science and Technology, China Agricultural University, Beijing 100193, China; Key Laboratory of Aquatic Botany and Watershed Ecology Wuhan Botanical Garden, Chinese Academy of Sciences, Wuhan 430070, China; State Key Laboratory of Marine Pollution, Department of Civil and Environmental Engineering, The Hong Kong Polytechnic University, Yuk Choi Road, Kowloon, Hong Kong 999077, China; Beijing Key Laboratory of Farmland Soil Pollution Prevention and Remediation, Department of Environmental Science and Engineering, College of Resources and Environmental Sciences, China Agricultural University, Beijing 100193, China

**Keywords:** microplastics, plastisphere, continental-scale, ecological patterns, homogenization

## Abstract

Microplastics alter niches of soil microbiota by providing trillions of artificial microhabitats, termed the “plastisphere.” Because of the ever-increasing accumulation of microplastics in ecosystems, it is urgent to understand the ecology of microbes associated with the plastisphere. Here, we present a continental-scale study of the bacterial plastisphere on polyethylene microplastics compared with adjacent soil communities across 99 sites collected from across China through microcosm experiments. In comparison with the soil bacterial communities, we found that plastispheres had a greater proportion of *Actinomycetota* and *Bacillota*, but lower proportions of *Pseudomonadota*, *Acidobacteriota*, *Gemmatimonadota*, and *Bacteroidota*. The spatial dispersion and the dissimilarity among plastisphere communities were less variable than those among the soil bacterial communities, suggesting highly homogenized bacterial communities on microplastics. The relative importance of homogeneous selection in plastispheres was greater than that in soil samples, possibly because of the more uniform properties of polyethylene microplastics compared with the surrounding soil. Importantly, we found that the degree to which plastisphere and soil bacterial communities differed was negatively correlated with the soil pH and carbon content and positively related to the mean annual temperature of sampling sites. Our work provides a more comprehensive continental-scale perspective on the microbial communities that form in the plastisphere and highlights the potential impacts of microplastics on the maintenance of microbial biodiversity and ecosystem functioning.

## Introduction

Plastic pollution is now one of the most conspicuous forms of global environmental change in the *Anthropocene* [1]. Globally, plastic production has skyrocketed in recent years, increasing from 1.5 million tons in 1950 to 367 million tons in 2020 [2], with an estimate 12 000 million tons of plastic waste ending up in landfills or the natural environment by 2050 [3]. Despite the remarkable convenience plastics provide to human lives and industrial activities, the unprecedented amount of poorly degrading plastic waste entering the environment challenges the functioning of global ecosystems. Over time, the plastic waste partially degrades because of environmental and biological stresses into microplastics [4-6]. Microplastics have become increasingly abundant throughout the biosphere, including marine, freshwater, atmospheric, and soil ecosystems, and are found even in the most remote parts of the planet [7-10]. The ubiquity of microplastics in the environment raises serious concerns about their potential and realized effects on ecosystems.

Microbial communities are particularly prone to be influenced by the increase in microplastics in the environment [11]. Microplastics can act as substrates, referred to as the “plastisphere,” that provide a variety of ecological niches for microorganisms [12]. Plastispheres are colonized by complex and dynamic microbial communities that have distinct composition compared with microbial assemblages in more natural surroundings [13-16]. Plastispheres may be a hot spot for horizontal gene transfer among microbes, potentially facilitating the dissemination of antibiotic resistance genes [17, 18]. Additionally, microbes in the plastispheres can play a strong role influencing biogeochemical cycling in the *Anthropocene* [19, 20]. Furthermore, microbial exposure may increase via the entry of microplastics into the food chain, leading to threats to biological safety and human health. Therefore, elucidating the microbial ecology of plastisphere would greatly contribute to the understanding and projecting of the ecological consequences of microplastic pollution.

Soils are the ideal settings for the acquisition and development of plastispheres as soils act as the long-term sink for microplastics [21, 22]. A handful of studies about soil plastisphere have estimated the ecological processes driving the microbial assemblages on microplastics. These studies suggest that polymer types as deterministic factors drive soil plastisphere microbiome assembly [16, 23, 24]. However, to date, most studies of the plastisphere focused only on one or a few specific soil types, which limit our ability to understand its variation across environments that differ in edaphic conditions. The roles of environmental variables in shaping plastisphere communities were generally overlooked in single soil studies, leading to an overestimation of the selective recruitment of microplastics. Thus, research with limited soils may not be sufficient to obtain a comprehensive view of the microbial assemblages in plastisphere. Furthermore, plastisphere communities are characterized by microbial species that are well adapted to the plastic properties (e.g. organic coating of the surface), which could potentially lead to microbial homogenization where similar communities form on microplastics across environmental conditions [25, 26]. However, at present, the hypothesis of microbial homogenization across plastispheres has not yet been directly tested because of the lack of large-scale observations across different soils in previous studies.

To explicitly test the hypothesis of whether plastispheres in the soil lead to homogenization of the microbial community across environments, we analyzed plastisphere and soil bacterial communities from 99 distinct soils along a large geographic and edaphic gradient across China. We compared the taxonomic and phylogenetic diversity of soil and plastisphere bacterial communities using high-throughput sequencing of 16S rRNA genes and estimated patterns of species compositional differences to examine the following questions: (i) Does the diversity and composition of bacterial communities differ between soils and the plastisphere? (ii) Is the composition among plastisphere communities across the sites more similar than that of the soil communities (i.e. is it more homogenous)? (iii) Do the underlying assembly processes differ between soil and plastisphere communities?

## Materials and methods

### Soil sampling and plastisphere incubation

To compare microbial communities in soil and the plastisphere across sites, we established microcosm experiment in soils from each of the 99 sites (Supplementary Fig. S1). Site characteristics, including location (latitude and longitude), climate (mean annual temperature and precipitation) (National Earth System Science Data Center [https://www.geodata.cn/]), soil texture (measured by the laser diffraction method [Topsizer Plus, China]), and other soil chemical features (see below for Methods), are presented in the Supplemental Information (SI). We collected soil samples from each site, which included seminatural forests, grasslands, or unreclaimed lands. To collect soil for the microcosm experiments and other analyses, we established a 10 × 10 m plot at each site, and collected five soil cores (depth of 0–20 cm and diameter 10 cm) that we then combined. We allowed the collected soil to air-dry at room temperature for 3 weeks and sieved it through a 2-mm mesh to remove plant debris and rocks.

To establish a plastisphere substrate for the microcosm experiment, we purchased polyethylene (PE) film (10 μm), cut it into 5 × 5 mm square fragments, and immersed it in hexane and methanol for 7 days to clean the sorbed chemicals. We then collected micro-fragments from the solvent and dried them in a fume hood before sterilizing them in a UV Clean Bench for 15 min prior to incubation. Soil microcosms for microbial growth were carried out in 1-l sterilized glass jars with ~500 g of soil. For the plastisphere incubation, we added 1000 microplastic items (mass concentration 0.04625%) into each soil microcosm based on our previous investigation [27, 28], and this microplastic level can be considered environmentally relevant [28]. We incubated the soil and plastisphere microcosms at 25°C for 180 days, and maintained water holding capacity of the soil at 60%. At the end of the experiment, we picked out microplastics from the jar, transferred them into a centrifuge tube, freeze-dried them, and then oscillated them at 2500 rpm to remove the surface-attached soil particles. We then used the microplastics without visible particles to investigate the plastisphere community. For soil analysis, we divided the soil sample into two subsamples and stored one at −80°C for community analysis and air-dried the other for chemical analyses.

### Soil chemical characterization

We determined a number of chemical variables in each soil sample, all of which are given in the supplemental information. We measured total carbon and total nitrogen using an elemental analyzer (Vario Macro Cube, Elementar, Germany). We measured soil pH and electrical conductivity (EC) in a 1:5 (m/v) soil suspension in deionized water. We measured dissolved organic carbon using a total organic carbon analyzer (Vario TOC, Elementar, Germany) after extracting from the soil using deionized water at a soil/water ratio of 1:10 (w/v). We measured the UV absorbance spectra of the DOM with a Shimadzu UV1900i UV–Vis spectrometer (Shimadzu, Japan). To do so, we calculated several spectral parameters (SUVA254, SUVA260, A253/A203, and E2/E3) using established methods [29, 30]. We determined soil ammonium (NH_4_^+^) and nitrate (NO_3_^−^) concentrations colorimetrically in soil extracts (1:5 (w/v), soil: 2 mol/L KCl solution) using the salicylic acid procedure of Mulvaney [31] and VCl_3_ procedure of Miranda *et al*. [32]. We measured available phosphorus in soil extracts (1:5 (w/v), soil: 0.5 mol/L NaHCO_3_ solution) using the molybdenum-blue method [33].

### DNA extraction and 16S rRNA gene analysis

To extract the total soil (~300 mg) DNA, we used a Mo Bio PowerSoil DNA Isolation Kit (Qiagen, Shanghai, China) following the manufacturer’s instruction. To extract plastisphere DNA, we placed ~200 pieces of micro-fragments into the extracting tube from Mo Bio PowerWater DNA isolation kit and followed the instructions (Qiagen, Shanghai, China). For microbial identification, we amplified the V3–V4 region of the 16S rRNA gene with the primer pair 338F (ACTCCTACGGGAGGCAGCA) and 806R (GGACTACHVGGGTWTCTAAT). We give the details of polymerase chain reaction in the supplemental information. We then sequenced the obtained amplicons in a 2 × 300-bp paired-end format using the Illumina Miseq platform at Majorbio BioPharm Technology Co. Ltd (Shanghai, China). The raw sequence data were deposited in Genome Sequence Archive (https://ngdc.cncb.ac.cn/gsa/s/9a3CC5Ys) under accession number PRJCA020079. The datasets during and/or analyzed during the current study are available from the corresponding author upon reasonable request.

To process the raw sequences, we used Quantitative Insights Into Microbial Ecology 2 (version 2020.2) with the DADA2 plugin [34]. After cutting the barcodes and primers, we trimmed the forward sequences to 280 base pairs and the reverse sequences to 200 base pairs. We then merged the sequences, denoised them, and clustered them into amplicon sequence variants (ASVs). We then used the SILVA reference database (Version 138) to assign taxonomic information to each representative ASV [35]. For the following analyses, we used data at the species level given the resolution of standard amplicon sequencing and the heterogeneity among samples.

### Statistical analysis

We quantified several parameters to assess the alpha diversity of soil and plastisphere communities, including extrapolated species richness (measured using the Chao1 metric), Shannon diversity, Pielou’s evenness, and Faith’s phylogenetic diversity, using QIIME2 [36]. We used Kruskal–Wallis rank-sum test to evaluate the difference between soil and plastisphere communities and considered a *P*-value of <.05 as statistically significant.

To evaluate compositional differences among soil and plastisphere communities across all sites, we calculated two values. The pairwise dissimilarity among communities and the dispersion—the multivariate distances between individual samples and the treatment centroids—within each group (soil communities and plastispheres) [37]. Communities with lower dispersion values have higher compositional similarity (i.e. greater homogenization) than those with higher dispersion, which are more heterogeneous in their compositions [38]. To visualize the difference between soil and plastisphere communities, we used principal coordinates analysis (PCoA) based on community dissimilarity. We calculated Bray–Curtis and Jaccard distances for taxonomic composition and weighted and unweight UniFrac dissimilarities for phylogenetic composition [39]. We used three different nonparametric multivariate statistical test (nonparametric multivariate analysis of variance [Adonis], analysis of similarity [ANOSIM], and multi-response permutation procedure [MRPP]) to test the differences in soil and plastisphere communities. We used a linear regression model between community similarity and geographic distance to explore the distance-decay pattern of microbial communities in the plastisphere and soil. We analyzed differences in dispersion between soil and plastisphere communities using the “betadisper” function in the “Vegan” R package [40].

We used a null model-based analyses of phylogenetic beta-diversity to estimate the potential contribution of different types of community assembly processes [41, 42]. Specifically, we applied the beta nearest taxon index (βNTI) coupled with the Bray–Curtis-based Raup–Crick (RC_bray_) metric, which quantifies the degree to which community structure differs from what would be expected by random (stochastic) changes. A value of |βNTI| > 2 indicates that the community assembly is likely to be governed primarily by deterministic processes, which can be divided into homogeneous (βNTI < −2) selection (i.e. communities more similar than expected by chance) and heterogeneous (βNTI > 2) selection (i.e. communities more different than expected by chance). On the other hand, |βNTI| < 2 suggests that community structure is determined mostly by chance, stochastic processes. This can be further partitioned by RC_bray_ values, where RC_bray_ < −0.95 suggests that higher dispersal rates or related processes lead to communities that are more similar, whereas RC_bray_ > 0.95 suggests that dispersal limitation leads to communities that are more different; when |RC_bray_| < 0.95, there are likely no dominant dispersal processes.

Next, we used the Sloan neutral community model [43] to predict the relationship between the frequency with which taxa occur in a set of local communities (detected proportion of taxon in individual soil or plastisphere community) and their abundance in the metacommunity (estimated by the mean relative abundance across all samples within plastisphere or soil communities). The overall fit to the neutral model is calculated based on the nonlinear least-squares fitting. A single free parameter, *m*, conveys the estimated migration rate; higher *m* values suggest less dispersal limited.

To further compare the difference between soil communities and plastisphere, we compared the interactions of taxa based on a network co-occurrence analysis of taxa with relative abundance > 0.01%. We used robust correlations with Pearson’s correlation coefficients *r* > 0.6 and false discovery rate-correlated *P*-values < .01 to construct networks. We calculated several network topology parameters, including average path length, network diameter, the clustering coefficient, and average degree [44]. We further compared the network stability of soil and plastisphere communities via the robustness metric, which describes the proportion of the remaining species in the network after random node removal [45], as well as, cohesion, an abundance-weighted, null model-corrected metric based on pairwise correlations across taxa to reflect the degree of cooperative behaviors or competitive interactions [46].

To assess the relationship between variation in environmental factors and variation in microbial communities in the soil and plastisphere, we used Procrustes analysis and Mantel tests [47]. Furthermore, we used a partial Mantel test to assess the relationship between community structure and each environmental variable; statistical significance was determined using 999 permutations [48]. To evaluate the relative importance of multiple factors contributing to the plastisphere microbial composition, we used the multiple regression on matrices (MRM) method that gives a measure of the rate of change in microbial community similar for variables when other variables were held constant [49]. To estimate the factors driving the distinction between plastisphere and soil communities, we calculated the Pearson correlations between the environmental factors versus the dissimilarities of soil and plastisphere communities, the differences in the relative abundance of the major phyla, and the variations in the alpha diversity indices. We estimated the importance of environmental variables to the distinction between compositional dissimilarities using a random forest analysis, with environmental variables serving as predictors [50].

## Results

### Composition and dissimilarity of soil and plastisphere communities

**Figure 1:**
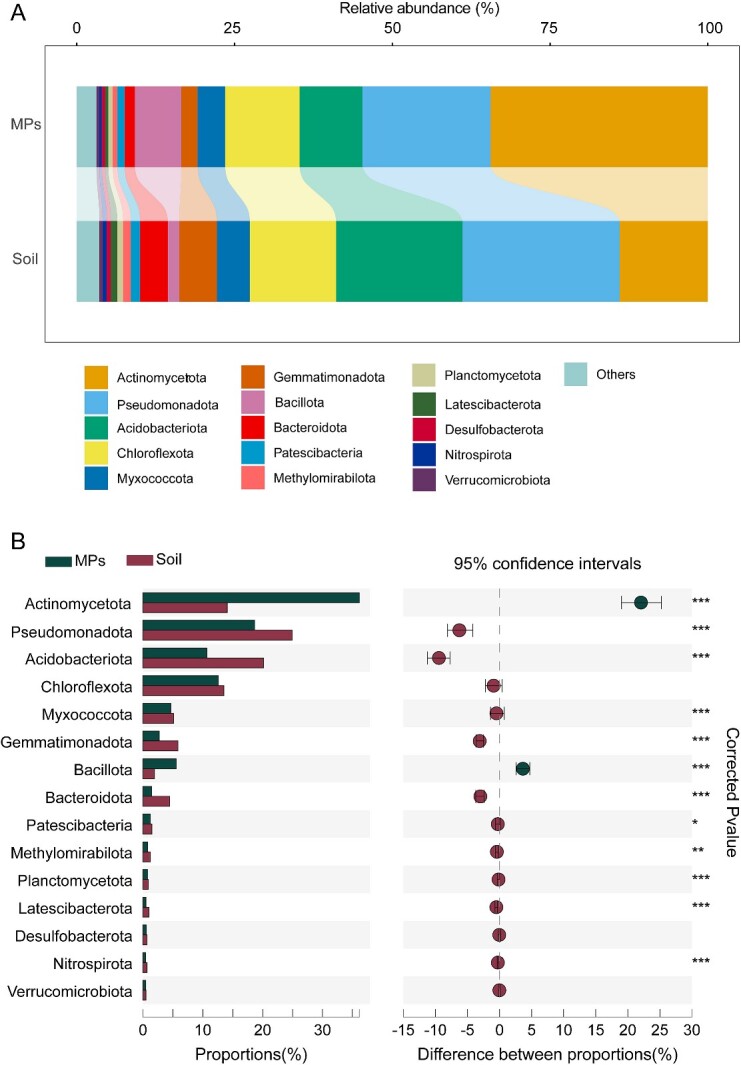
(A) The bacterial community composition at the phylum level in soil (*n* = 99) and plastisphere (*n* = 99); the unclassified bacteria were included in the others; (B) comparison of the relative abundance of the main phyla in soil and plastisphere; Bars represent mean ± SD of the proportion (%); cycle symbols represent differences between soil and plastisphere samples; statistical significance is based on Kruskal–Wallis rank-sum tests; ^*^P < .05, ^*^^*^P < .01, ^*^^*^^*^P < .001. MPs, plastisphere bacterial communities; soil, soil bacterial communities.

We found that the richness of bacterial communities was statistically (Kruskal–Wallis rank-sum test, *P* < .05) greater for plastisphere, whereas the evenness was relatively lower (Kruskal–Wallis rank-sum test, *P* < .05), potentially suggesting the compositional bias of plastisphere (Supplementary Fig. S2). When we analyzed species compositional differences, we found that the plastisphere harbors microbiomes that are distinct from adjacent soil ecosystems. At the phylum level, the relative abundances of *Actinomycetota* and *Bacillota*, on average, were 34.4% and 7.4% in the plastisphere, respectively (Fig. 1 and Supplementary Fig. S3), which were significantly higher (Kruskal–Wallis rank-sum test, *P* < .001) than those in soil microbiomes (13.9% and 1.8%, respectively). Other dominant phyla, including *Pseudomonadota*, *Acidobacteriota*, *Gemmatimonadota*, and *Bacteroidota*, were more abundant in soil bacterial communities. At the class level, we found that the relative abundances of *Actinobacteria*, *Thermoleophilia*, *Acidimicrobiia*, *Bacilli*, and *Chloroflexia* were higher in the plastisphere compared with the soil (Supplementary Fig. S4).

**Figure 2:**
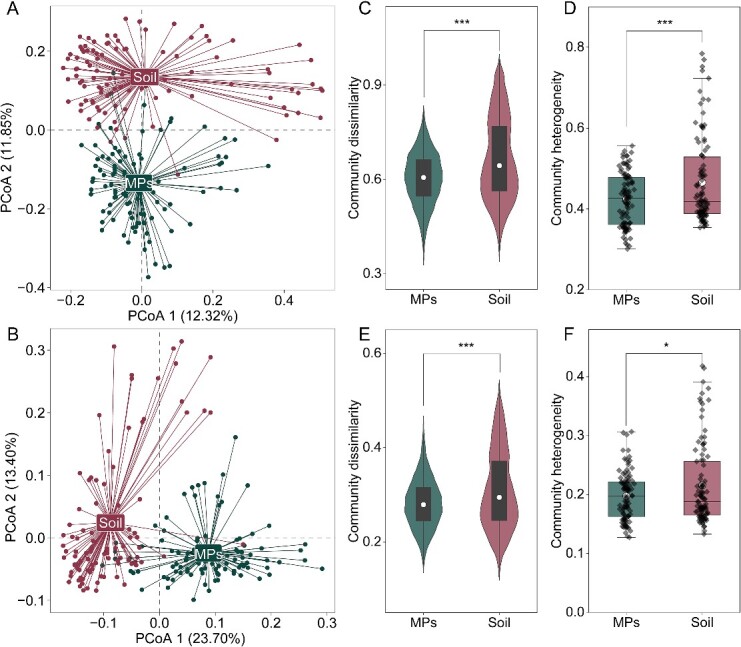
(A) PCoA on the basis of Bray–Curtis distance revealing the dissimilarity between soil and plastisphere samples; (B) PCoA on the basis of weight UniFrac distance revealing the dissimilarity between soil and plastisphere samples; (C) violin plots showing the within-group (plastisphere or soil) community dissimilarity on the basis of Bray–Curtis distance; the white cycle symbols represent medians; the tops and bottoms of the boxes show the 75th and 25th percentiles, respectively; (D) the community composition heterogeneity of microorganisms in soil and plastisphere samples; boxes include median and 75th/25th percentile of the distances to the group centroid derived from betadisper (vegan R package) on the basis of Bray–Curtis; (E) violin plots showing the within-group (plastisphere or soil) community dissimilarity on the basis of weighted UniFrac distance; The white cycle symbols represent medians; the tops and bottoms of the boxes show the 75th and 25th percentiles, respectively; (F) The community composition heterogeneity of microorganisms in soil and plastisphere samples. Boxes include median and 75th/25th percentile of the distances to the group centroid derived from betadisper (vegan R package) on the basis of weighted UniFrac; asterisks indicate significant differences between different groups based on permutational multivariate analysis of variance tests. ^*^P < .05, ^*^^*^P < .01, ^*^^*^^*^P < .001.

We visualize the differentiation between plastisphere and soil communities using PCoA based on Bray–Curtis dissimilarity and found that the plastisphere samples were generally separated from the soil communities (Fig. 2A). When we used three complementary nonparametric multivariate statistical tests (Adonis, ANOSIM, and MRPP), we found that the bacterial community structure was quite distinct between the plastisphere and soil communities (Supplementary Table S1). When our analyses incorporated phylogenetic differences based on the weighted UniFrac distance, we also found a clear separation between microplastic and soil samples (Fig. 2B). In addition, we found that the plastisphere tended to host more homogeneous bacterial communities than those found in soil ecosystems (*P* < .001) (Fig. 2C–F). That is, we found a greater dissimilarity (*P* < .001) in the bacterial community composition across the soil samples than across the corresponding plastisphere.

**Figure 3:**
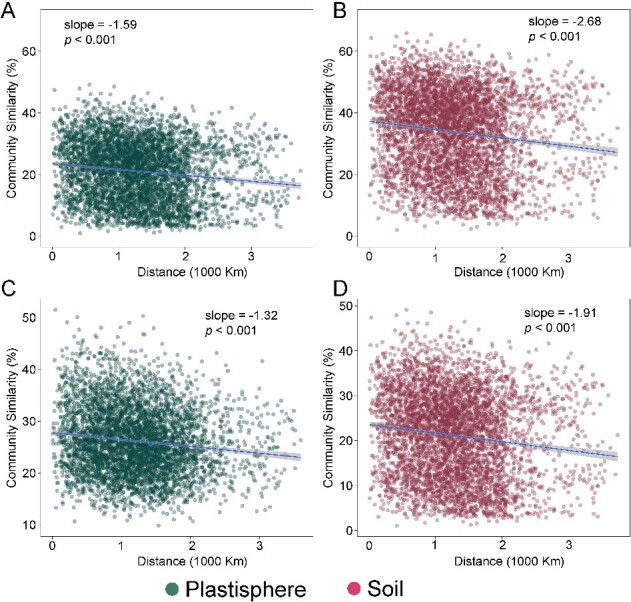
(A) The DDR based on Bray–Curtis distance revealing the relationship between plastisphere community similarity and geographical distance; (B) the DDR based on Bray–Curtis distance revealing the relationship between soil community similarity and geographical distance; (C) the DDR based on weighted UniFrac distance revealing the relationship between plastisphere community similarity and geographical distance; (D) the DDR based on weighted UniFrac distance revealing the relationship between soil community similarity and geographical distance; the solid lines indicate significant relationships.

To further compare the difference in community variation between soil and plastisphere communities, we estimated the distance–decay relationship (DDR) on both Bray–Curtis and weighted UniFrac distances. We found negative DDRs across both soil communities (slope = −2.68 for Bray–Curtis distance and −1.91 for weighted UniFrac) and plastisphere communities for both metrics (slope = −1.59 for Bray–Curtis distance and −1.32 for weighted UniFrac), suggesting the community similarity decreases as the geographical distance increases (Fig. 3). Furthermore, the DDR slopes for soil communities were significantly steeper than the slopes for plastisphere communities, indicating the spatial turnover rate of the plastisphere would be lower than that observed in natural soil habitats.

### Assembly processes and coexistence in soil and plastisphere microbiome

**Figure 4:**
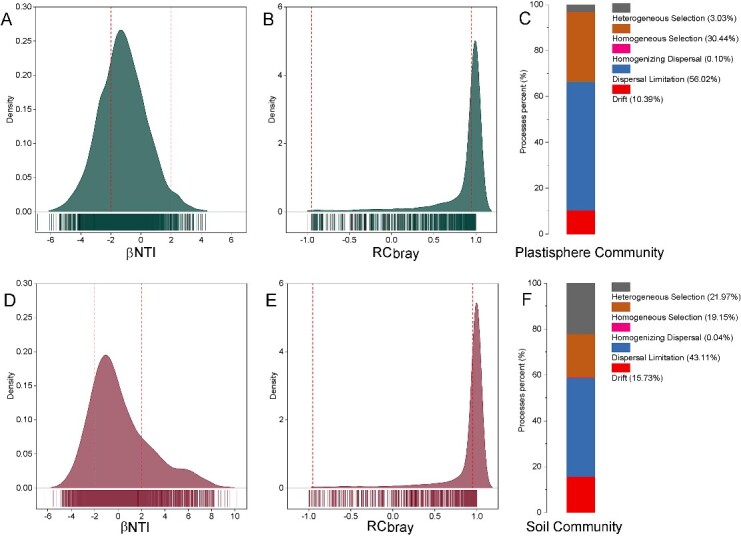
The null model results of the plastisphere and soil bacterial communities; (A) the distribution of standardized phylogenetic turnover among plastisphere sample; (B) the distribution of standardized taxonomic turnover among plastisphere sample; (C) the relative importance of different ecological processes to the plastisphere assembly; (D) the distribution of standardized phylogenetic turnover among plastisphere sample; (E) the distribution of standardized taxonomic turnover among plastisphere sample; (F) the relative importance of different ecological processes to the plastisphere assembly; the vertical dashed lines mark the positions of −2 and 2 in panels A and D, and −0.95 and 0.95 in panels B and E.

**Figure 5:**
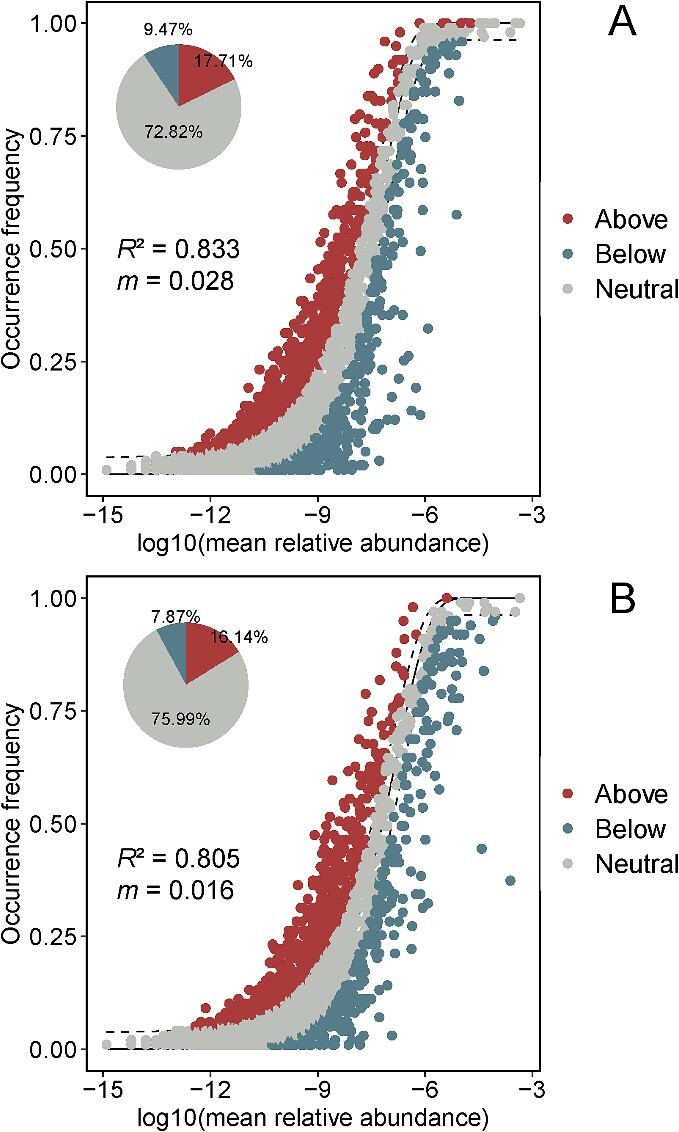
Fit of the neutral model. The predicted occurrence frequencies for the plastisphere (A) and the soil microbial communities (B), respectively; species that occur more frequently than predicted by the model are shown in red, whereas those that occur less frequently than predicted are shown in blue; dashed lines represent 95% confidence intervals around the model prediction; the pie charts represent the relative distribution of predicted species.

Using the null model (Fig. 4), we found that stochastic processes accounted for 66.53% of the patterns of compositional structure across the soil communities and 58.88% of the compositional structure across the plastisphere samples. With the patterns consistent with stochastic processes, we found that dispersal limitation was more likely driving patterns for both soil microbial (explaining 56.02% of the variation) communities and the plastisphere (explaining 43.11% of the variation). We found a negligible contribution of homogeneous dispersal to the observed patterns. Compared with the soil communities, our observed patters are consistent with a lower relative importance of deterministic processes in the plastisphere. In particular, the relative importance of patterns consistent with homogeneous selection was higher in the plastisphere communities (30.44%) compared with the soil samples (19.15%). When we parameterized the Sloan neutral model to (Fig. 5), we found that both plastisphere and soil communities fit the neutral community model, but the degree of fit was relatively higher in microplastic samples (*R*^2^ = 0.833) than that in the soil communities (*R*^2^ = 0.805). Estimates of the migration parameter (*m*) were low for both communities, but slightly higher in the plastisphere communities.

**Figure 6:**
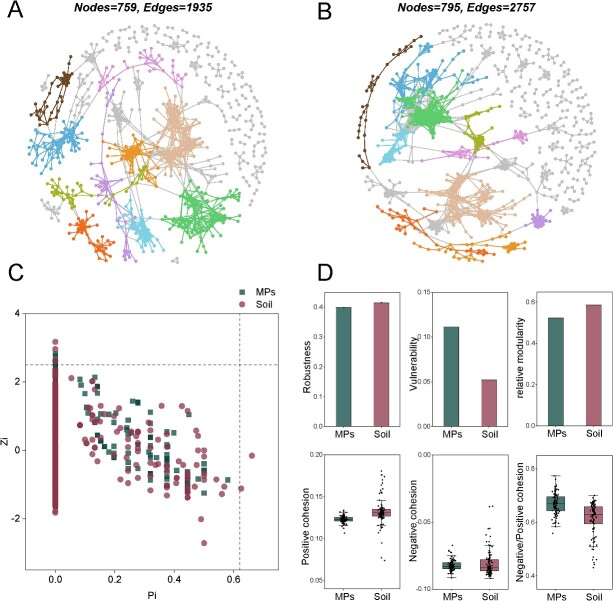
The network of microbial community; (A) visualization of constructed molecular ecological network for plastisphere communities; top 10 large modules are shown in different colors, and smaller modules are shown in gray; (B) visualization of constructed molecular ecological network for soil communities; top 10 large modules are shown in different colors. (C) the within-module connectivity (Zi) and among-module connectivity (Pi) of each node in the networks. Module hubs (Zi ≥ 2.5, Pi < 0.62), connectors (Zi < 2.5, Pi ≥ 0.62), and network hubs (Zi ≥ 2.5, Pi ≥ 0.62) are identified as the keystone nodes; the vertical dashed line marks the position of 2.5 and the horizontal dashed line marks the position of 0.62; (D) the indices indicating the stability and complexity of networks.

When we generated co-occurrence networks for soil and plastisphere communities (Fig. 6), we found fewer nodes and edges in the plastisphere (759 nodes and 1935 edges) network than in the soil network (795 nodes and 2757 edges), as well as a lower average degree, average clustering coefficient, and relative modularity. We also quantified the degree of community complexity using the index of cohesion, where positive cohesion can reflect the cooperative behaviors among species, whereas negative cohesion is associated with competitive interactions. We found higher positive cohesion values (average 0.132) in the soil microbial network relative to the plastisphere network (average positive cohesion value, 0.123). In addition, we observed that the absolute values of negative cohesion were higher in the plastisphere network. To compare the robustness of soil microbial and plastisphere networks, we simulated random species extinction and calculated the resistance to node loss of the networks. We found lower robustness values in the plastisphere network compared with the soil network. The robustness value was 0.393 in the plastisphere network after 50% of species were removed, and the value was 0.415 in the soil microbial network. Finally, we found that the network vulnerability (the maximum decrease in network efficiency when a single node is deleted from the network) was also higher in the plastisphere network (0.111) compared with the soil microbial network (0.052).

### Drivers of microbial community dissimilarities between soil and plastisphere samples

**Figure 7:**
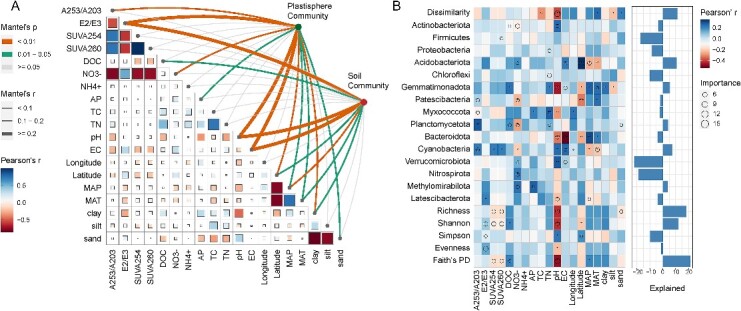
(A) Correlations of the plastisphere and soil community structure (Bray–Curtis distance) with the soil and climate variables; edge width corresponds to the Mantel’s *r* value, and the edge color denotes the statistical significance; pairwise correlations of the variables are shown with a color gradient denoting Pearson’s correlation coefficients; (B) contributions of the soil and climate variables to the differences (alpha-diversity, Bray–Curtis dissimilarity, and relative abundances of the main phyla) between plastisphere and soil communities based on correlation and random forest model; circle size represents the variables’ importance (i.e. percentage of increase of mean square error calculated via random forest model); colors represent Spearman’s correlations.

We found that variation in the species composition of both plastisphere and soil communities was highly correlated to those of the environmental variables using both Procrustes and Mantel tests (Supplementary Fig. S4). Partial Mantel correlations between microbial communities and each of the environmental factors indicated that several variables were associated with compositional variation in both the plastisphere and soil communities (Fig. 7A). Importantly, although some of the significant variables were the same between both community types (E2/E3, pH, EC, latitude, MAP, MAT, and clay), other variables were unique in their associations with plastisphere or soil communities (Fig. 7A). Overall, pH and EC were the strongest correlates of taxonomic compositions in the plastisphere. Our MRM analyses revealed the relative importance of the environmental factors to the plastisphere community structure (Supplementary Table S2). The overall MRM model was significant (*P* < .001) and explained 22.3% of the variation (i.e. *R*^2^ = 0.223). The DOM features, including SUVA254 and SUVA 260, had the largest regression coefficients, but the results were not statistically significant. Other DOM features, i.e. A253/A203 and E2/E3, were significantly contributed to the plastisphere composition. In addition, the soil pH and EC were also the significant drives in shaping the microbial communities on the microplastics. Clear turnovers existed in taxonomic composition of plastisphere communities with the changes in soil pH and EC (Supplementary Fig. S5).

We compared the community dissimilarities between soil communities and plastisphere for each pairwise set of soil samples. In addition, the differences in the relative abundance of the dominant phyla and alpha-diversity indices of each pairwise set were also calculated. In Fig. 7B, we illustrate the relationship between several environmental variables and the difference between plastisphere and soil communities in several community variables. Among the soil chemical properties, we found that the soil pH was among the most important, showing a strong correlation with the dissimilarities between soil communities and plastisphere, distinction in the alpha diversity indices, and differences in relative abundances of most phyla. Other variables, including MAT, soil carbon, and the proportion of sand in the soil explained some of the difference between the communities in the plastisphere and soil.

## Discussion

Overall, results from our study indicate that: (i) microbial community structures on plastispheres diverged from that on soil microbial communities, (ii) plastispheres harbored more similar microbiomes to one another across diverse conditions than the adjacent soils, potentially because of the greater homogeneous selection, and (iii) soil pH and other variables play an important role in determining the assembly of microbiota in the plastisphere.

Prior to our study, it was unclear whether the plastisphere exhibits distinct microbial communities in soil. In aquatic environments, planktonic microbe communities that dominate the water column strongly diverge from those able to associate and form biofilms [51-53], and thus it is not surprising that the plastisphere communities are distinct compared with the planktonic communities. In soil, microbes can form supracellular structures in biofilms comprised of surface associated microbial cells embedded in hydrated extracellular polymeric substances [53], which has some similarities with the structure of the plastisphere. As a result, the majority of bacterial groups in the plastisphere and soils should be similar, which we observed. However, the divergence in community structure we observed between soil communities and the plastisphere is likely because of the selection of microplastics. The surface hydrophobicity of microplastic particles is higher than that of the soil particles, which induces the microplastics to sorb dissolved organic matters (DOM) from the soil and recruit copiotrophic bacteria such as, *Actinomycetota* and *Bacillota* [16, 54, 55]. As a consequence of the distinct community composition in the plastisphere compared with their embedded soil samples, together with the vast amounts of plastic litter in the environment, the microbial biomass of plastisphere is likely to be substantial with important effects on ecosystem functions [56-58].

Compared with variation among soil microbial communities, we found that the plastisphere exhibited more homogeneous communities across the sampling sites. It is increasingly recognized that anthropogenic-induced impacts, such as because of climate change, nutrient addition, or urbanization, can reduce environmental heterogeneity and increase homogenization of natural communities, threatening ecosystem biodiversity and functionality [59-61]. Our findings provide novel evidence that microplastics, as a factor of global change, may promote the homogenization of microbial communities. The surface properties of microplastics can predispose them to be colonized by copiotrophic species, whereas oligotrophic species are known to be more abundant in nutrient-poor environments that are rarer in the plastisphere [62]. This deflective selection may lead to microbial homogenization on microplastics. Indeed, our null model results, as well as those from our parameterization of the neutral community model, suggested that the microbial communities in the plastisphere were less constrained by environment than the microbial communities in the soils. A possible explanation for this is that microplastics could be unique habitats because of the formation of biofilms, outweighing the filtering because of environmental factors [63]. Furthermore, the relative contribution of homogeneous selection for the plastisphere community was relatively high, possibly because the microplastics served as a consistent substrate across the different soil samples that led to the recruitment of microbial communities with little variation.

Our results revealed that environmental factors, in particular soil organic carbon content and pH, were associated with the differences in community composition between the soil and the plastisphere. In soil environments with rich organic carbons, the differences in nutrient conditions between microplastics and soil would be small, leading to weak effects of plastisphere selection. Conversely, microplastics in the carbon-poor soils would act as the external strong sorbents to sorb DOM, which could lead to the enrichment of available carbon around microplastics and induce strong microbial recruitment [64, 65]. Likewise, one reason soil pH may contribute to our observed dissimilarities is because of the positive relationship between soil organic matter and pH in the range of 4–8 [66, 67]. That is, soil with lower pH values have less organic carbon [68], which would be favored by oligotrophic microorganisms with low carbon use efficiency. Furthermore, the composition of soil DOM varied following the changes in soil pH values [69]. Because aliphatic-like and proteins/amino sugar-like compounds are often negatively correlated with soil pH [70], the DOM in acidic soils should be more labile and available and more easily sorbed by microplastics. Furthermore, acidic conditions may promote the aging of microplastics, which potentially facilitate the release of polymer additives and oligomers [71, 72]. As a result, a unique habitat with a relatively high level of nutrients and energy would be formed around the microplastic particles in acidic soils, leading to higher dissimilarities between soil and plastisphere communities.

Whether anthropogenic chemical contaminants drive biodiversity loss in ecosystems has received increasing attention in recent years [59, 73, 74]. Our work indicates that the plastisphere is characterized by higher proportions of copiotrophic microbe species. More similar microbiomes are harbored among microplastics across a broad environmental gradient relative to their adjacent soil samples. The findings provide the first continental-scale evidence of the homogenization of plastisphere, potentially suggesting that microplastics, as a global change factor, can threaten global biodiversity. Even though we found an important role for stochastic processes associated with community structure of the plastisphere, the homogeneous recruitment of microplastics, because of their hydrophobic surfaces, may contribute to this homogenization. Our study extends the knowledge of the ecology and biogeography of plastic-associated microbial communities, which provides a deeper understanding of the interaction between anthropogenic chemical pollution and biodiversity loss under global change.

This study may still have some limitations. First, only bacterial community was investigated, other microbiomes including archaea, fungi, and protists were not determined. Whether these microbial communities would be distinct from those in natural soil ecosystem and whether they are more homogeneous are currently unknown. Even in the studies with single soil, very few studies have comprehensively evaluated a wide range of functional and taxonomic groups in plastisphere. Considering that the fungal hyphae can spread on microplastics and that the protists may exhibit potential predation or symbiosis relationships with bacteria, these microbial taxa in plastisphere may display distinct patterns. Furthermore, we simply used PE microplastics as the substrates for microbial colonization. In the real environment, microplastics may originate from different products with various polymers and morphologies. These microplastic idiosyncrasies may potentially contribute to the microbial assemblages in plastisphere. Additionally, *ex situ* microcosms were employed in the current study, *in situ* incubation or fielding sampling would be better to generate a synoptic view of microbial ecology in the plastisphere. Regardless of these limitations, our study helps disentangle how the man-made materials introduced by human civilization are altering the natural microbial ecology in the *Anthropocene*.

## Conflicts of interest

None declared.

## Funding

The National Natural Science Foundation of China (Grant Number 42377381).

## Data availability

The raw sequence data were deposited in Genome Sequence Archive (GSA; https://ngdc.cncb.ac.cn/gsa/s/9a3CC5Ys) under accession number PRJCA020079. The datasets during and/or analyzed during the current study are available from the corresponding author upon reasonable request. R scripts for key analyses in this study are available at https://github.com/watertimes/continental-scale-plastisphere.

## Supplementary Material

SI_r1_ycad012

table_S1_ycad012
