# Supplementary material for: Homogenization of bacterial plastisphere community in soil: a continental-scale microcosm study
Source: ISME Commun. 2024 Jan 10;4(1):ycad012. doi: 10.1093/ismeco/ycad012 (PMC10848224; doi:10.1093/ismeco/ycad012)
Supplement: SI_r1_ycad012 [file si_r1_ycad012.docx]

Supporting Information for:

**Homogenization of bacterial plastisphere community in soil: A** **continental-scale microcosm study**

Yuanze Sun^1^, Mochen Wu^1^, Siyuan Xie^1^, Jingxi Zang^1^, Xiang Wang^2,*^, Yuyi Yang^3^, Changchao Li^4^, Jie Wang^1,*^

^1^ Beijing Key Laboratory of Farmland Soil Pollution Prevention and Remediation, College of Resources and Environmental Sciences, China Agricultural University, Beijing 100193, China

^2^ Key Laboratory of Arable Land Conservation (North China), College of Land Science and Technology, China Agricultural University, Beijing, 100193, China

^3^ Key Laboratory of Aquatic Botany and Watershed Ecology Wuhan Botanical Garden, Chinese Academy of Sciences, Wuhan 430070, China

^4^ Department of Civil and Environmental Engineering and State Key Laboratory of Marine Pollution, The Hong Kong Polytechnic University, Kowloon, Hong Kong, China

*Corresponding Authors

Xiang Wang, [wangxiang@cau.edu.cn](mailto:wangxiang@cau.edu.cn)

Jie Wang, [jiewangcau@cau.edu.cn](mailto:jiewangcau@cau.edu.cn)

Table S2 Significance tests of the soil communities and plastispheres

|  | Adonis | | ANOSIM | | MRPP | |
| --- | --- | --- | --- | --- | --- | --- |
|  | R2 | *p* | r | *p* | δ | *p* |
| Taxonomic diversity  Bray-Curtis  Jaccard  Phylogenetic diversity  Weighted UniFrac  Unweighted UniFrac | 0.0884  0.0592  0.1578  0.0346 | 0.0001  0.0001  0.0001  0.0001 | 0.3355  0.3355  0.4291  0.1529 | 0.0001  0.0001  0.0001  0.0001 | 0.6341  0.7703  0.2953  0.5663 | 0.0001  0.0001  0.0001  0.0001 |

Table S3 The MRM results reveal the relative importance of the environmental factors to the plastisphere community structure.

| Environmental factors | coefficient | *p*-value |
| --- | --- | --- |
| A253/A203  E2/E3  SUVA254  SUVA260  DOC  NO_3_^-^  NH_4_^+^  AP  TC  TN  pH  EC  MAP  MAT  clay  silt  sand | 0.0324  0.0301  0.6205  -0.6670  -0.0011  0.0223  0.0003  0.0049  -0.0061  0.0116  0.0214  0.0212  0.0119  0.0044  0.0145  0.0065  -0.0058 | 0.0037  0.0003  0.0953  0.0751  0.8858  0.0071  0.9800  0.4728  0.6463  0.3713  0.0048  0.0099  0.1522  0.5945  0.1516  0.4402  0.5636 |
|  | Total explainable portion (*R*^2^) | *p*-value |
|  | 0.2232 | 0.0001 |


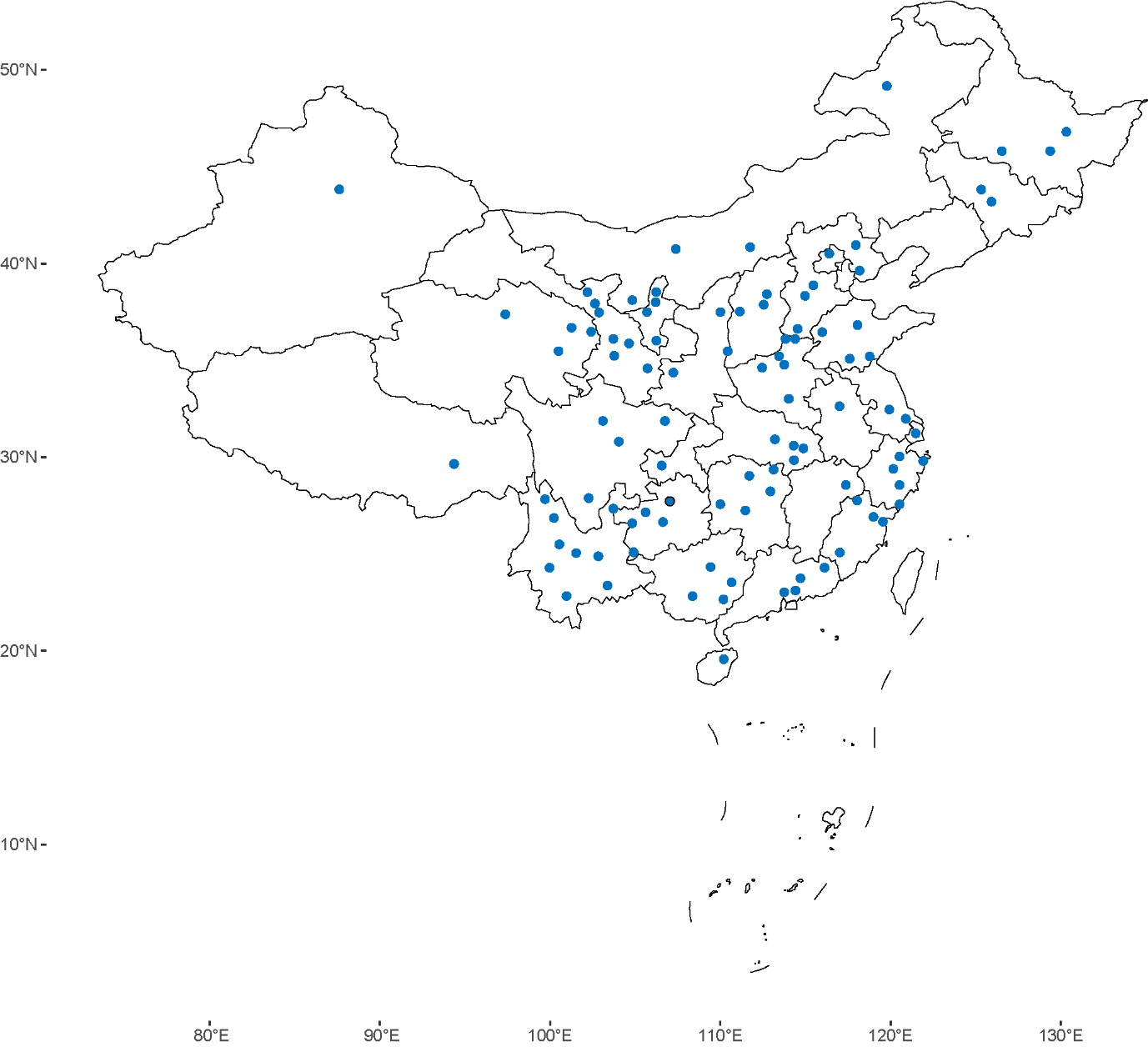


Figure S1. The location of sampling sites


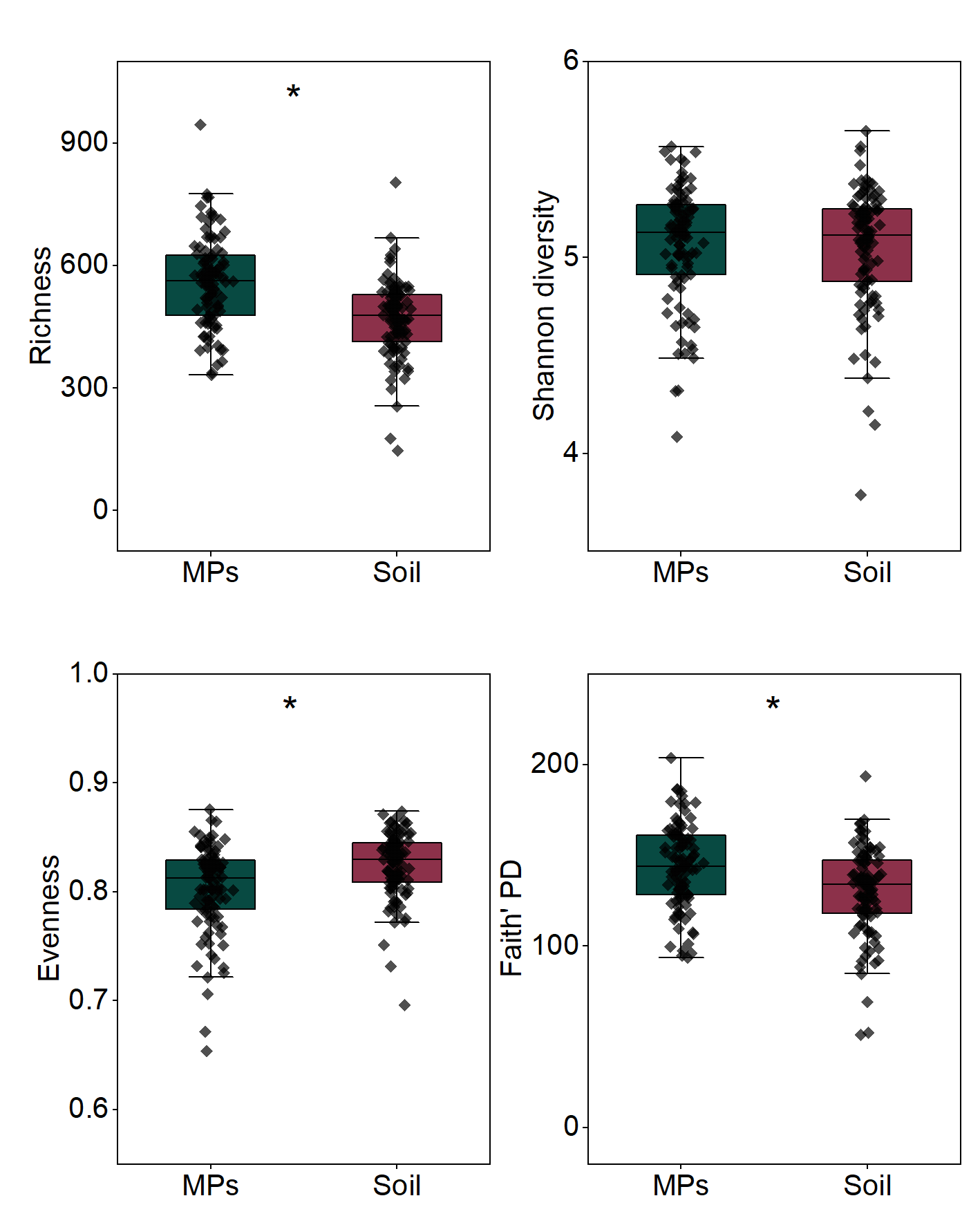


Figure S2. The alpha diversity of soil and plastisphere communities. Statistical significance is based on Kruskal-Wallis rank-sum tests; * *p* < 0.05, ** *p* < 0.01, *** *p* < 0.001.


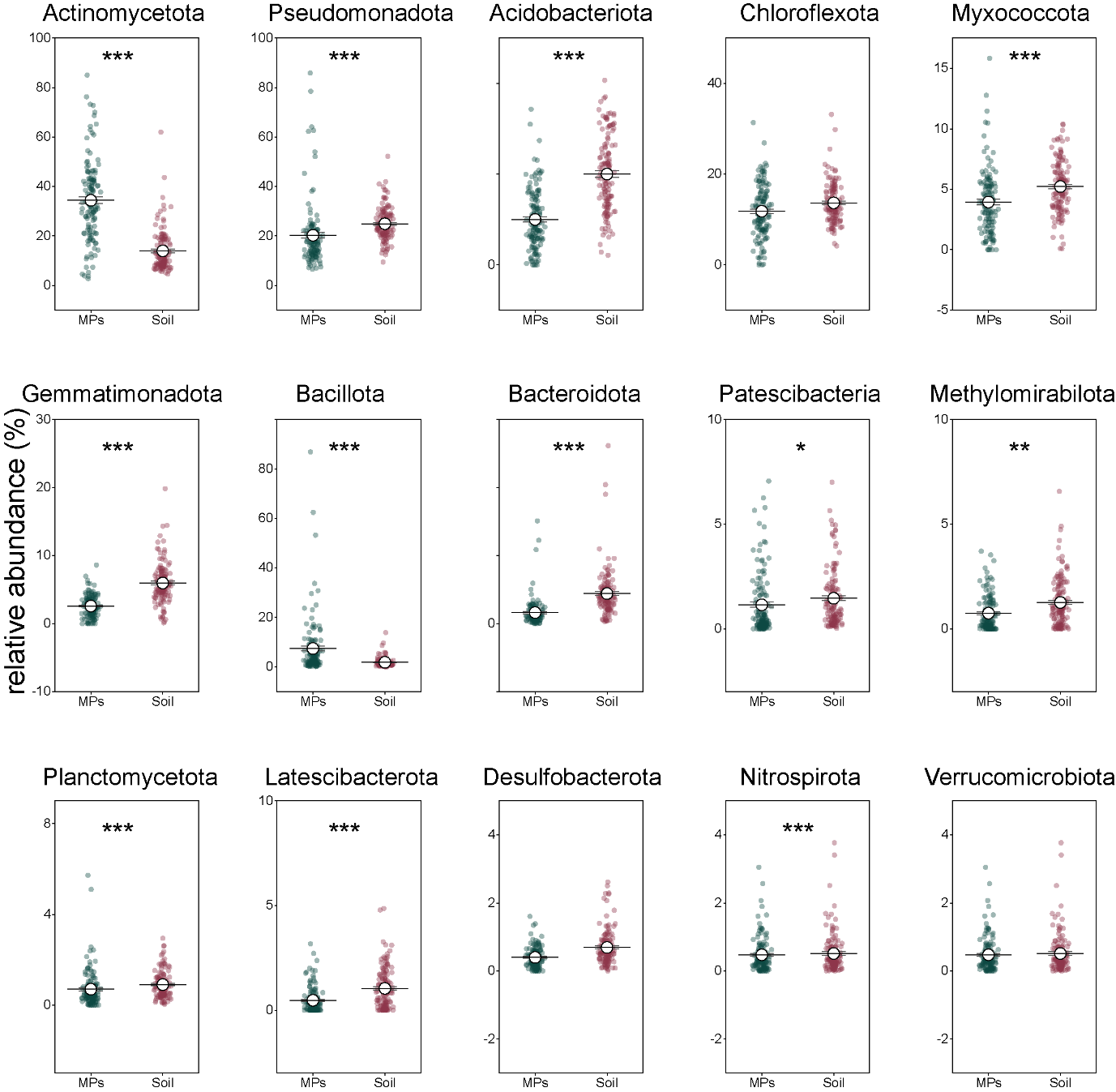


Figure S3. The relative abundance of the main phyla in soil and plastisphere bacterial communities. Statistical significance is based on Kruskal-Wallis rank-sum tests; * *p* < 0.05, ** *p* < 0.01, *** *p* < 0.001.


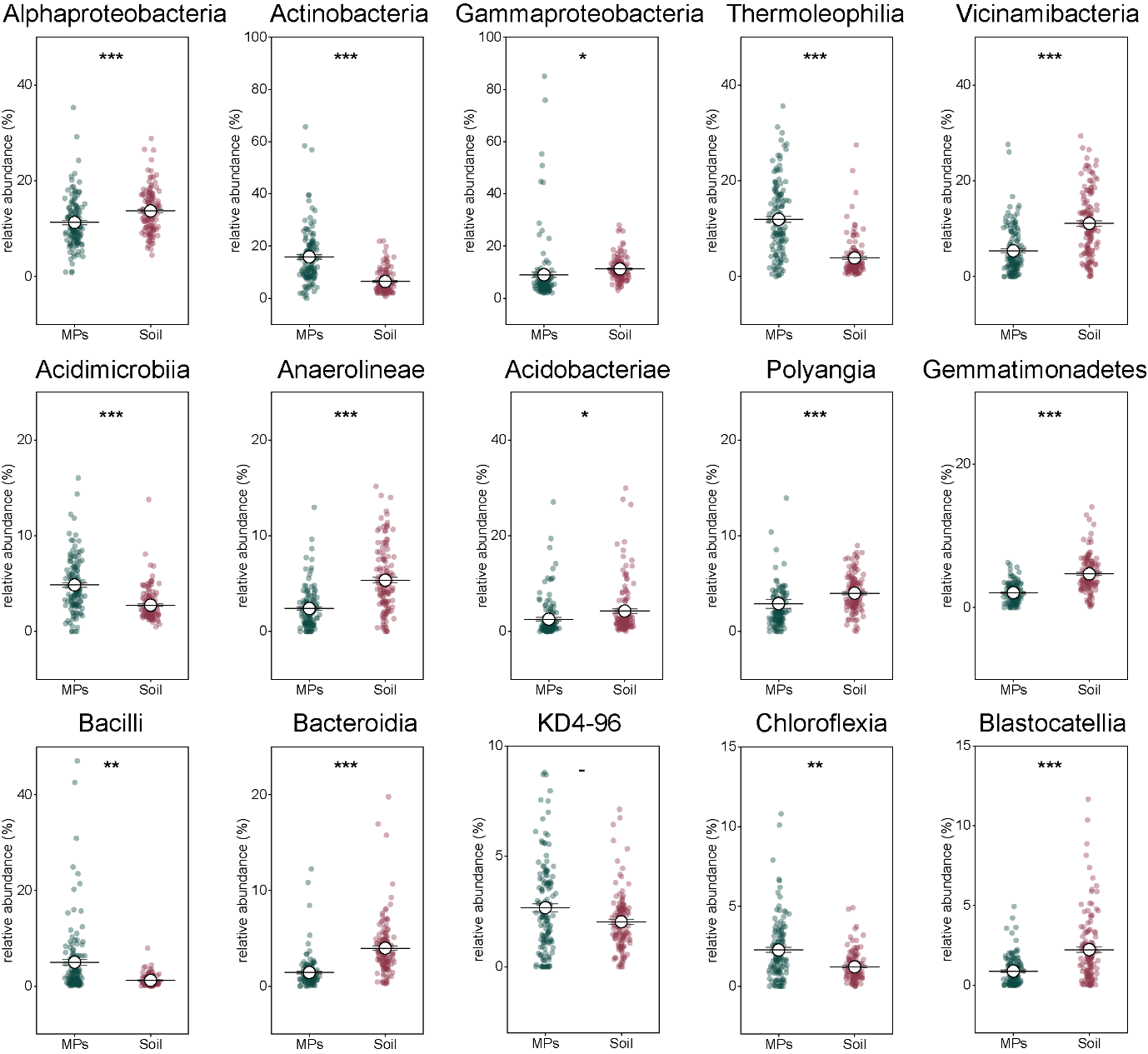


Figure S4. The relative abundance of the main class in soil and plastisphere bacterial communities. Statistical significance is based on Kruskal-Wallis rank-sum tests; * *p* < 0.05, ** *p* < 0.01, *** *p* < 0.001.


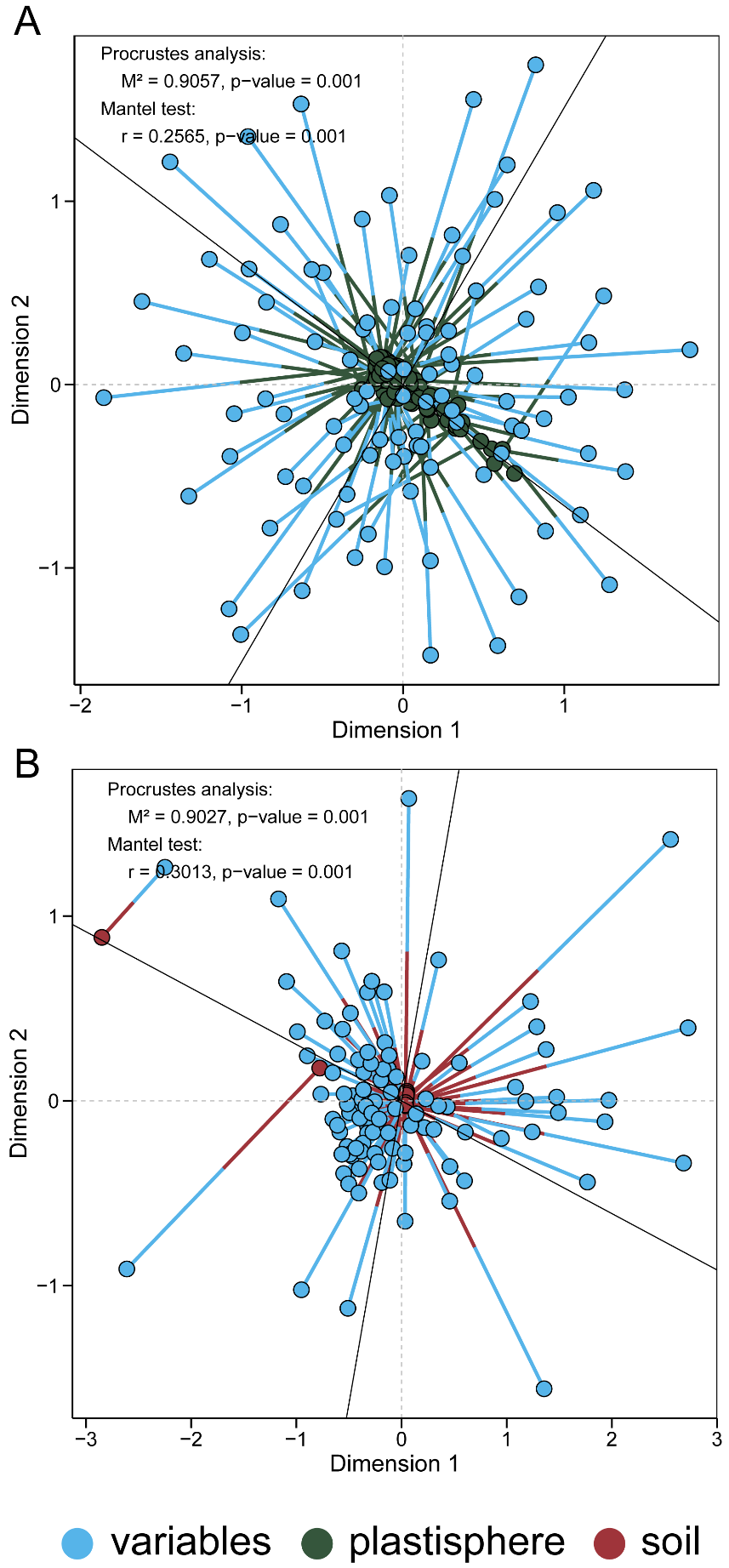


Figure S4. The results of Procrustes analysis and Mantel test between environmental variables and the plastisphere (A) and between environmental variables and the soil communities (B).


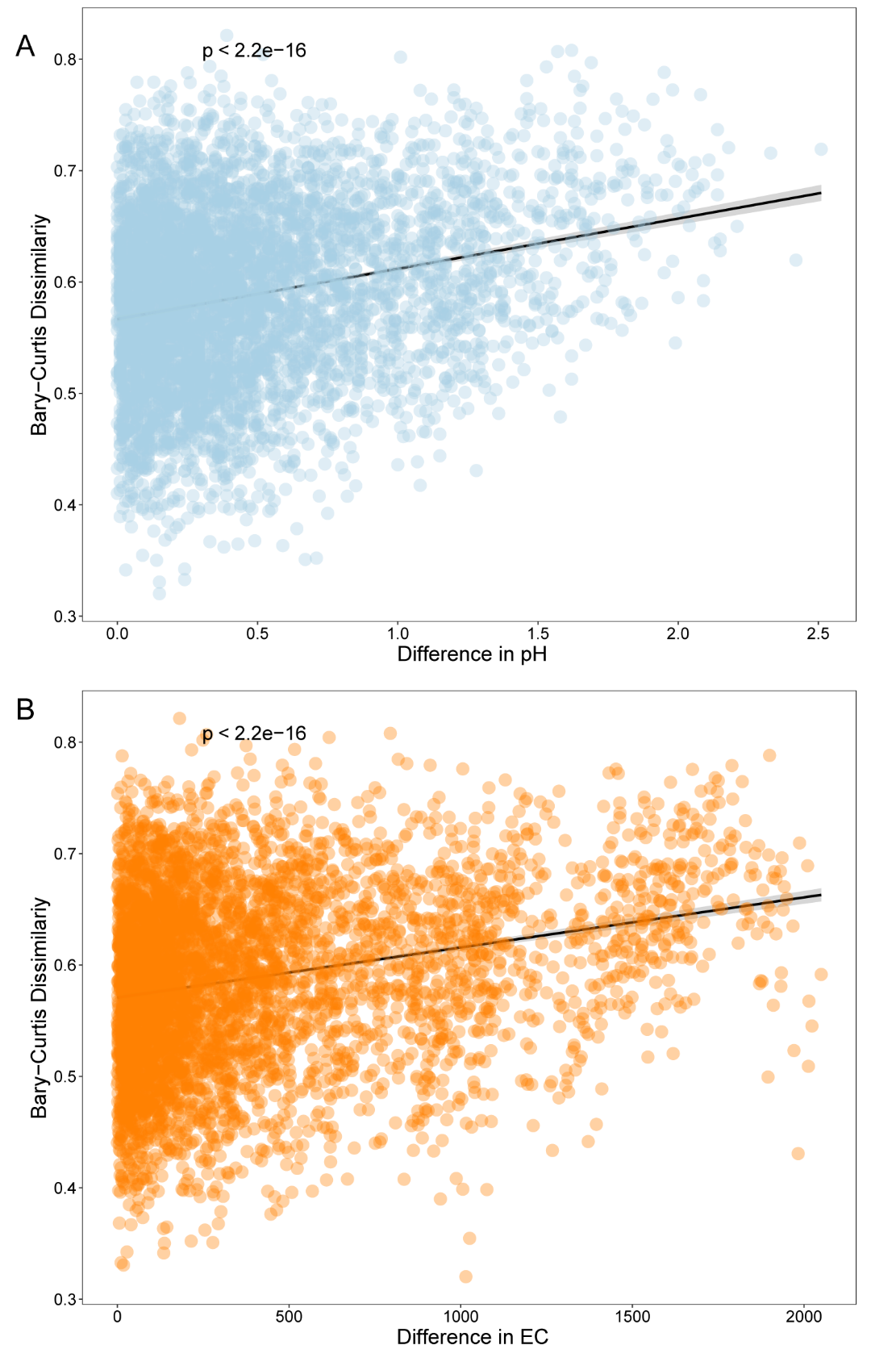


Figure S5. The turnover between plastisphere communities and pH (A) and EC (B) differences among the sampling sites.
